# Enabling women to access preferred methods of contraception: a rapid review and behavioural analysis

**DOI:** 10.1186/s12889-021-12212-7

**Published:** 2021-11-27

**Authors:** Abimbola A. Ayorinde, Felicity Boardman, Majel McGranahan, Lucy Porter, Nwamaka A. Eze, Anna Sallis, Rosanna Buck, Alison Hadley, Melissa Ludeke, Sue Mann, Oyinlola Oyebode

**Affiliations:** 1 Warwick Medical School, University of Warwick, Coventry, CV4 7AL UK; 2 Public Health England, Wellington House, Waterloo Road, London, SE1 8UG UK; 3 Teenage Pregnancy Knowledge Exchange, University of Bedfordshire, Luton, LU1 3JU UK

**Keywords:** Contraception, Women, Access, Barriers, Facilitators, Intervention, Choice

## Abstract

**Background:**

Many pregnancies in the UK are either unplanned or ambivalent. This review aimed to (i) explore barriers and facilitators to women choosing and accessing a preferred method of contraception in the United Kingdom, and (ii) identify opportunities for behavioural interventions based on examination of interventions that are currently available nationally.

**Methods:**

Three databases were searched, and experts contacted to identify grey literature for studies presenting barriers and facilitators to women choosing and accessing a preferred method of contraception, conducted in the UK and published between 2009 and October 2019. Information on barriers and facilitators were coded into overarching themes, which were then coded into Mechanisms of Actions (MoAs) as listed in the Theory and Techniques Tool. National interventions were identified by consulting stakeholders and coded into the Behaviour Change Wheel. The match between barriers/facilitators and intervention content was assessed using the Behaviour Change Wheel.

**Results:**

We included 32 studies and identified 46 barrier and facilitator themes. The most cited MoA was Environmental Context and Resources, which primarily related to the services women had access to and care they received. Social Influences, Beliefs about Consequences (e.g., side effects) and Knowledge were also key. The behavioural analysis highlighted four priority intervention functions (Modelling, Enablement, Education and Environmental Restructuring) that can be targeted to support women to choose and access their preferred method of contraception. Relevant policy categories and behaviour change techniques are also highlighted.

**Conclusions:**

This review highlights factors that influence women’s choices and access to contraception and recommends opportunities that may be targeted for future interventions in order to support women to access preferred contraception.

**Registration:**

Protocol was registered with PROSPERO (an international database of prospectively registered systematic reviews in health and social care) in December 2019, CRD42019161156.

**Supplementary Information:**

The online version contains supplementary material available at 10.1186/s12889-021-12212-7.

## Background

About 16% of pregnancies in the UK are classed as unplanned while 29% are classed as ambivalent [1]. Approximately 76% of women in the United Kingdom (UK) use contraception but discontinuation and change in use of method of contraception contributes to high rates of unplanned pregnancy [2]. For example, a prospective study showed that within 1 year 5% of women stop using their contraception and 12% switch methods of contraception [3]. The study also demonstrated that women who discontinue contraception during the follow-up period and those who switched to a different method of contraception have a risk of unplanned pregnancy that is almost as high as those who do not use any contraception at all [3]. Women who are dissatisfied with, or neutral about, their contraceptive methods are up to seven times more likely to discontinue or switch contraceptive methods compared to those who are satisfied [4]. Ensuring that women who do not want to be pregnant are aware of contraception, know how to access it and that women are satisfied with their choice of contraception could significantly improve uptake and continuity of use and ultimately reduce the rate of unintended pregnancy. Understanding factors that influence the decision to choose and access contraception is important to facilitate the provision of preferred contraception, which is compatible with the user’s lifestyle to encourage uptake and facilitate continuity of use [5].

As well as understanding the factors that enable and hinder women choosing a preferred contraceptive (i.e., barriers and facilitators), it is important to understand which intervention strategies can be used to target these factors. The Behaviour Change Wheel (BCW) is a tool that can help to achieve this. It was developed to help build, characterise and evaluate behaviour change interventions and consists of the Capability Opportunity Motivation model of behaviour (COM-B, which can be used to assess the barriers and facilitators to a behaviour), a list of intervention functions (IFs), and a list of policy categories that can be used to support the implementation of those interventions [6]. The tool links these three components together, and consequently allows intervention developers to link the key barriers/facilitators influencing a specific behaviour to the intervention strategies most likely to support behaviour change.

The COM-B model at the heart of the BCW maps onto the Theoretical Domains Framework, a list of “domains” that describe different kinds of influence on behaviour (e.g., Knowledge, Social Influences, Environmental Context and Resources) [7]. More recently, the Theory and Techniques Tool (TaTT) has been developed, which contains an extended list of these domains, in this case called “Mechanisms of Action” (or MoAs) [8]. The TaTT provides another opportunity to link barriers/facilitators of a behaviour to intervention strategies, by providing an interactive online tool that links MoAs to specific behaviour change techniques (BCTs).

Together, these tools can be used to group influences on behaviour together (i.e., by coding them into MoAs) and identify IFs, policy categories and BCTs to achieve change. Once these appropriate intervention strategies have been identified for any given behaviour, they can also then be used to evaluate the existing interventions that are currently being provided. This allows policymakers to assess whether there are any missed opportunities in current programmes [9].

This rapid review was part of a larger Public Health England (PHE) funded project to examine ways to improve reproductive outcomes in the UK. The rapid review presented in this paper was designed to explore factors that enable or hinder women to choose and access a preferred method of contraception in the United Kingdom (UK). Using the tools described above, we characterised these barriers/facilitators into MoAs so that we could identify appropriate intervention strategies for targeting them (in this case, IFs as listed in the BCW). We also identified and examined the contents of the current available interventions that aim to support women to choose and access a preferred method of contraception, in order to identify missed opportunities for interventions. The goal was to make recommendations that would improve current interventions and inform future interventions.

## Methods

### Information sources and search strategy

In October 2019 we searched three electronic databases; MEDLINE, PsycINFO and CINAHL. We used a wide range of synonyms and MeSH terms relating to behaviour, barriers, facilitators or healthcare (such as, behaviour, choice, barriers, facilitators, health care, self-help, service utilisation) combined with terms relating to birth control (such as, contraceptive, contraception, family planning). The search strategy developed in collaboration with an Academic Support Librarian at Warwick Medical School and a Senior Information Scientist from PHE. We used a validated UK search filter developed by the National Institute for Health and Care Excellence (NICE) to limit the MEDLINE search to studies conducted in the UK (we could not use this filter for the other two databases because the filter was validated for use in MEDLINE only) [10]. The MEDLINE search strategy is available in Additional file 1.

We contacted relevant experts to identify grey literature, national interventions, and additional studies that we may have missed by our searches. To do this, we sent emails out through professional networks to those in various organisations such as the Faculty of Sexual and Reproductive Healthcare (FSRH), National Health Service, Family Nurse Partnership, Department of Health and Social Care, local governments, Royal College of Midwives, National Reproductive Health Systems Leadership Forum, PHE and asked them to cascade amongst their networks as well. The FSRH also published a notice on their website. Interventions were considered national interventions if they were accessible nationally regardless of whether they were provided by local or national organisations. Interventions included websites with useful information/educational materials for women and training interventions aimed at healthcare professionals. Interventions targeted at local populations were not included.

### Study selection

We imported citations into EndNote software and removed duplicates. Titles and abstracts were screened by one reviewer, and a 10% sample was double screened by a second reviewer. Two reviewers independently screened full-texts of potentially relevant articles against pre-specified selection criteria (Table 1) and disagreements were resolved by discussion between the two reviewers. The wider team (which includes the PHE Sexual and Reproductive Health Lead and Project Steering Group) was consulted in instances where consensus could not be reached between the two reviewers. We included both qualitative and quantitative studies in women reporting barriers and facilitators for women to choose and use a preferred form of contraception. Only studies conducted in the UK and published between 2009 and October 2019 were included. We also selected articles presenting interventions aimed at supporting women to choose or access appropriate and preferred methods of contraception in order to examine their contents. We excluded studies on the use of contraceptives for purposes other than contraception. For example, the use of hormonal contraceptives to regulate menstruation and the use of condoms to prevent sexually transmitted infections. We also excluded studies that focus mainly on emergency contraception and abortion as well as articles such as protocol papers, editorials, comments and conference abstracts.

**Table 1:** Inclusion and exclusion criteria

Inclusion criteria	Exclusion criteria
*Population*: women (adolescents and older) *Outcomes*: exploring barriers to and facilitators for women choosing and accessing an appropriate and preferred method of contraception. *Study type*: quantitative and qualitative studies of any design published in the last 10 years. Setting: We restricted to studies conducted in the UK, to ensure that the findings are relevant to UK settings. We also selected articles presenting interventions aimed at supporting women to choose or access appropriate and preferred methods of contraception in order to examine their contents for intervention mapping of the behavioural analysis.	Studies assessing the clinical effectiveness of specific contraceptives. Studies on the use of contraceptives for purposes other than contraception. For example, hormonal contraceptives to regulate menstruation; condoms to prevent sexually transmitted infections. Studies that focussed on emergency contraception and abortion. Studies from outside the UK. Articles lacking methods required for quality appraisal (such as conference abstracts).

### Data extraction

We designed and pre-piloted a data extraction tool using Microsoft Excel to record: study identification (author, year), study design, aims, location and population, sample size, age and ethnicity of participants. We used NVivo 12 software to facilitate the coding of data on barriers and facilitators. Due to the volume of evidence and time constraints, one reviewer conducted data extraction and a random sample of extractions was reviewed by a second team member.

### Quality assessment

We used the Mixed Methods Appraisal Tool (MMAT) for quality assessment of studies [11]. The MMAT includes five quality appraisal criteria for each of five study designs including qualitative studies, randomized controlled trials, non-randomized studies, quantitative descriptive studies, and mixed methods studies. Quality assessment was performed by one reviewer and checked by a second reviewer.

### Data synthesis and behavioural analysis

Data on barriers and facilitators were synthesised descriptively to provide a broad overview of the current evidence. We grouped text excerpts describing similar concepts of barriers and facilitators together into themes. We conducted a behavioural analysis to further examine the themes identified, characterise the content of available interventions and identify opportunities for intervention improvement. To do this, we grouped the barrier and facilitator themes into the MoAs listed within the TaTT [8]. This exercise was performed by one reviewer but checked with the wider team throughout. As described above, MoAs are theoretical constructs that represent different kinds of influence on behaviour (e.g., Knowledge, Social Influences, Environmental Context & Resources) [8]. We listed the barrier/facilitator themes against all the MoAs they were linked to (themes could be linked to more than one MoA) and for each MoA, we recorded the number of themes as well as the corresponding number of associated references. We then ranked the MoAs based on (i) the number of studies that identified a barrier or facilitator linked to that MoA and (ii) the number of themes identified that link to that MoA. We considered MoAs which were mentioned in at least 25% of studies and were associated with a minimum of two barrier/facilitator themes as key MoAs for prioritisation.

**Fig. 1:**
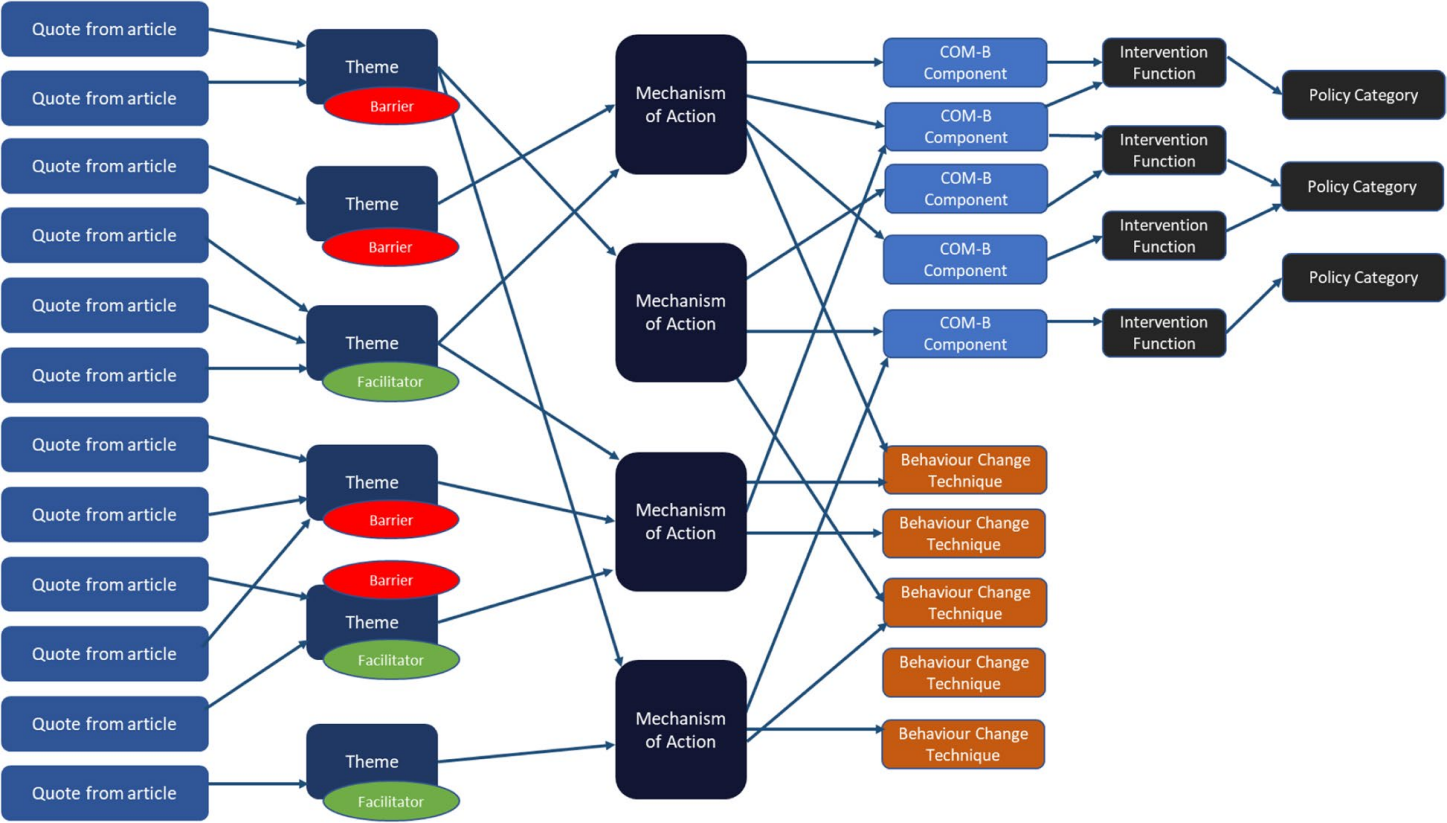
Behavioural Analysis Schema. Quotes from included articles were coded into themes and these were linked to Mechanism of Actions (MoAs) listed within the Theory and Techniques Tool (TaTT). We used TaTT matrix to identify Behaviour Change Techniques that are theoretically linked to each of the identified MoAs. All MoAs were also linked to component of COM-B. We identified Intervention Functions and Policy Categories that were theoretically linked to the MoAs using the Behaviour Change Wheel

To identify appropriate intervention strategies, we used the BCW to link MoAs to IFs (see PHE, 2020 for a full list and examples of IFs or “intervention types”). This was done by linking the MoAs to the COM-B model of behaviour, which then links on to the list of IFs within the BCW. Some of the MoAs (i.e., those that had already appeared in an earlier framework called the Theoretical Domains Framework; see Introduction) were already linked to the appropriate component of COM-B (i.e., capability, opportunity and/or motivation) model in the literature [12]. Any additional MoAs that had not yet been linked to COM-B were linked through an interim mapping exercise overseen by Professor Susan Michie, which was conducted in advance of a definitive mapping of MoAs to COM-B (to be conducted). Once MoAs had been linked to COM-B components, we could then identify which IFs and policy categories were theoretically linked to the key MoAs representing our barriers and facilitators. We also used the TaTT matrix to identify BCTs that are theoretically linked to each of the identified MoAs. A schema illustrating the behavioural analysis process is presented in Fig. 1.

We then examined the contents of national interventions aimed at helping women choose a preferred method of contraception and coded them into IFs and policy categories. We then highlighted IFs and policy categories that are not included in the available interventions but were theoretically linked to key MoAs as missed opportunities that could be targeted for future interventions.

### Stakeholder consultations

We consulted with both the public (women) and professionals as part of this review. PHE had a specific interest in two populations with increasing rate of unintended pregnancies; black and minority ethnic groups, and women over 30 years of age. Therefore, we selected an organisation with a diverse reach for our public engagement. We worked with Foleshill Women’s Training (FWT) – A Centre for Women (www.fwt.org.uk), a Coventry based women only organisation that particularly supports women from marginalised and vulnerable groups, such as migrant, refugee and asylum seekers. We held two meetings at FWT in January and February 2020. We invited women from any ethnic group regardless of gravidity and parity. Twelve women attended each meeting, including British women from various ethnic backgrounds and migrant women from Iran, Afghanistan, the Caribbean, Pakistan, Sri-Lanka and India. For many of the women, English was not their first language and staff at FWT translated for those who did not speak English. We explained the aims of the project and discussed our emerging findings. We requested the women’s opinions on the topics. Each meeting was facilitated by two research team members - one led the engagement whilst the other took notes. Findings from the public engagement are not included in the behavioural analysis but supported validation and interpretation of our findings. We worked closely with a project steering committee and used our networks to identify professional stakeholders (individuals and organisations) from whom we requested input. We also requested literature and details of known interventions from professionals as highlighted in the previous section.

The review protocol was registered with PROSPERO (an international database of prospectively registered systematic reviews in health and social care) in December 2019, CRD42019161156 [13]. We used the Preferred Reporting Items for Systematic Reviews and Meta-Analyses (PRISMA) checklist to guide the reporting of this review [14] (Additional File 2).

## Results

**Fig. 2:**
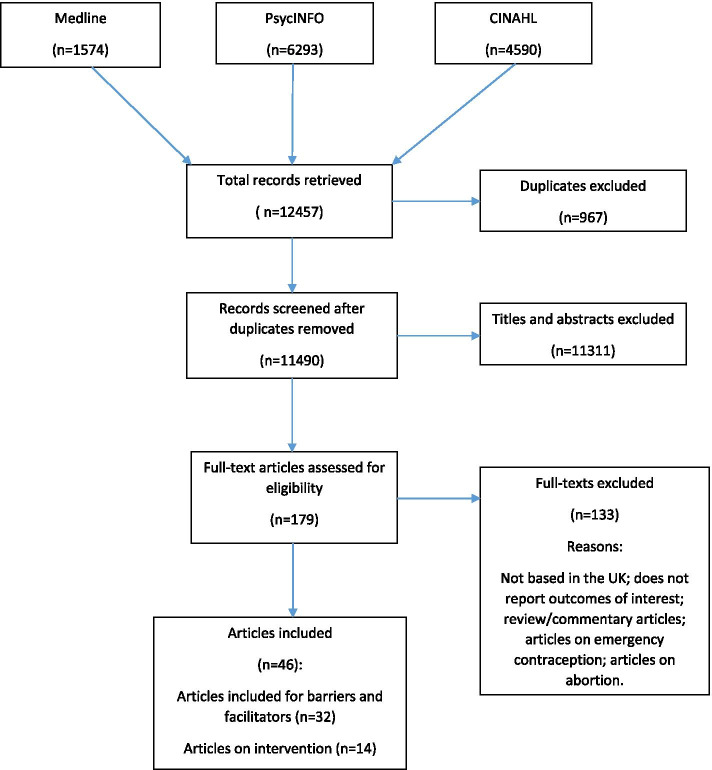
PRISMA flow diagram for study selection

The search identified 12,457 citations and duplicates were removed. After screening 11,490 titles and abstracts 179 citations were retained for full-text screening. Forty-six studies relating to contraception met the inclusion criteria (Fig. 2). Thirty-two of these presented barriers to and facilitators for women choosing and using a preferred form of contraception (Additional File 3), while 14 were used for extracting information on relevant interventions only. Of the 32 included studies, 13 involved qualitative methods, 12 cross sectional surveys, six mixed methods and one was an audit.

### Quality assessment

Twenty of the thirty-two papers met at least 70% of quality criteria, of which 13 met all the relevant quality criteria (Additional File 4). One article was an audit and did not fulfil the initial screening question of the MMAT tool and so we could not perform quality assessment on it [15].

### Barriers and facilitators

We grouped the barriers and facilitators reported in the included studies into 46 themes. Additional File 5 presents the list of themes and examples of text associated with the themes. The most frequently identified themes are women’s personal experience/other people’s experience (*n* = 10 studies), concerns about side effects (*n* = 9) and healthcare professionals not providing sufficient information about contraception to women (*n* = 9). Women also frequently reported concerns relating to mechanism of the contraceptive methods or procedure of fitting them (*n* = 7), effects on menstruation (*n* = 6) and poor information or knowledge about various contraception methods (*n* = 6). Pressure or influence of young men and violence, advice from healthcare professionals, effectiveness of the method at preventing pregnancy and the lack of involvement of women with learning disabilities in decision making together with an absence of accessible information resources for them were reported in five studies each. The remaining themes were identified in fewer studies.

### Key mechanisms of action

We coded the 46 themes into 17 of the possible 26 MoAs (Additional File 6). The number of themes coded into each MoA varied from one (in each of Behavioural Cueing, Behavioural Regulation and Subjective Norms) to 16 (in Environmental Context and Resources). Eight MoAs met the predetermined criteria to be included as key MoAs (i.e. they were associated with at least 25% of the included studies and two themes). Themes associated with the key MoAs are listed in Table 2. We identified IFs that are theoretically linked to the key MoAs (Table 2). The IFs that are linked to the largest number of themes within the key MoAs were Enablement (associated with 41 themes), Environmental Restructuring (41 themes), Modelling (34 themes) and Education (32 themes).

**Table 2:** List of Key Mechanism of Actions (MoAs), the associating themes and intervention functions

MoA (COM-B)	Themes associated with the MoA	Intervention Functions linked to COM-B^**a**^
Environmental Context and Resources (Physical opportunity)	(i) Advice from health care professionals (ii) Advice from informal sources (iii)Age limiting method of choice [sterilisation] (iv)Contraception not a priority [for homeless] due to competing priorities/Lack of stability and transient lifestyle due to homelessness (v) Cost (vi)Underlying medical condition (diabetes) limiting choice (vii) Health care professional’s lack of knowledge (viii) Women’s lack of understanding of the UK context (ix)Language barrier and cultural misunderstanding (x)Women with learning disability not being involved in decision making or lack accessible information resources (xi)Not being registered with GP (xii)Perceived resistance by health care professionals to remove implant (xiii)Unwelcoming healthcare setting (xiv)Accessibility of location and time (including organisation of health service) (xv)Easy availability of method (xvi)Support by someone (such as key workers) for contraception appointment (for women with intellectual disability)	T, R, ER, En
Social Influences (Social opportunity)	(i)Advice from informal sources (ii)Embarrassment (iii)Personal experience and other people’s experience (iv)Pressure or Influence of young men and violence (v)Religious background (vi)Unwelcoming healthcare setting (vii)Expectations of use and the influence of others (viii)Partners perceived willingness [to use condom] (ix)Relationship with health professionals (x)Support by someone (such as key workers) for contraception appointment (for women with learning disability)	R, ER, M, En
Beliefs about Consequences (Reflective motivation)	(i) Anticipated emotional cost of accessing services for women with drug problems (ii)Concern about adding extra chemicals or hormones to the body/Perception that hormonal contraceptives are unnatural (iii)Concern about side effects (iv) Effect on menstruation (v)Mechanism of the contraceptive methods or procedure of fitting them (vi)Protects against sexually transmitted diseases (vii)Comfort or convenience of method (viii)Effectiveness of method at preventing pregnancy (ix)Perceived positive benefit [predicts intention to use long-acting reversible contraception]	Ed, P, M
Knowledge (Psychological capability)	(i) Women’s lack of understanding of the UK context (ii)Language barrier and cultural misunderstanding (iii)Poor information or knowledge about various birth control methods (iv Health care professionals not providing sufficient information about contraception to women (v)Not knowing where to get help or advice (vi)Misconceptions about IUD (vii)Real life experience of seeing an IUD (viii)Knowledge of where to access services (ix)Low perceived value of undergoing intervention as barrier for women with drug problems	Ed
Attitude towards the behaviour (Automatic motivation and reflective motivation)	(i)Concern about adding extra chemicals or hormones to the body/Perception that hormonal contraceptives are unnatural (ii)Personal experience and other peoples experience	Ed, P, I, C, T, ER, M, En
General Attitudes / Beliefs (reflective and automatic motivation and capability)	(i)Trustworthiness of information source (ii)Lackadaisical attitude, acceptance of pregnancy or feel pregnancy is not a problem (iii) Being ‘in the moment’ (iv)Concern about adding extra chemicals or hormones to the body/Perception that hormonal contraceptives are unnatural (v)Preference for female GP (vi)Religious background or grounds	Ed, P, I, C, T, ER, M, En,
Perceived susceptibility / vulnerability (Automatic motivation and reflective motivation)	(i) Anticipated emotional cost of accessing services for women with drug problems (ii)Issues relating to mechanism of the contraceptive methods or procedure of fitting them (iii)Misconceptions about IUD	Ed, P, I, C, T, ER, M, En
Values (Automatic motivation, reflective motivation and social opportunity)	(i) Lackadaisical attitude, acceptance of pregnancy or feel pregnancy is not a problem (ii)Protects against sexually transmitted diseases (iii)Low perceived value of undergoing intervention as barrier for women with drug problems	E, P, I, C, T, R, ER, M, En

^a^Key: *Ed* Education, *P* Persuasion, *I* Incentivisation, *C* Coercion, *T* Training, *ER* Environmental restructuring, *M* Modelling, *En* Enablement, *R* Restriction

### Findings from public engagement

Several barriers to and facilitators for choosing and accessing contraception were raised by women at FWT. These include:

- Language barriers limiting the ability to access or understand information available
- Poor internet access and/or information technology (IT) skills limiting the ability to access information
- The perceived need to be assertive to get what they want
- The stigma around contraception as a topic for discussion
- Lack of information contraception during education in their home countries
- Prioritisation of the wishes of male partners/ lack of support from partner. Many women expressed that they consider their partners wishes before anything else: If their partner does not want them to talk to anyone, they would not; If he does not approve of contraception, or a particular type of contraception, they would not use it.
- Misunderstanding and/or misinformation from health professionals (for example a woman reported that a GP had told her specific forms of contraception were not compatible with breast-feeding, which is not the case- unclear whether the GP had definitely given this wrong information or whether the woman had misunderstood).
- Lack of trust/ potential to receive inaccurate information from social influences. “Person you trust (e.g. friend, partner) might not give you the right information”. (This was in response to who might interpret for them during consultations for contraception).

Most of these themes map to the MoAs determined to be key by the findings of the literature review, although some of the barriers raised map into additional MoAs: “Skills” (relating to barriers such as poor language and IT skills) and “Emotion” (relating to barriers such as stigma, prioritisation of partners wishes and lack of trust to receive accurate information from social influences). This suggests these additional MoAs may also be important for this more marginalised and vulnerable group of women.

### Missed opportunities for intervention

We collated 15 interventions, which we regarded as nationally active, with sufficient information to code. We coded the 15 interventions into five out of the nine IFs (Additional File 7): Education (n_number of interventions_ = 9), Environmental restructuring (*n* = 8), Enablement (*n* = 6), Restriction (*n* = 2) and Persuasion (*n* = 1). These cover three of the four priority IFs (Enablement, Environmental Restructuring and Education). No national intervention linked to Modelling. We highlighted the IFs that are theoretically linked to the key MoAs via COM-B but for which there were no corresponding national interventions and they were therefore identified as missed opportunities (Table 3).

**Table 3:** Missed opportunities for interventions to support women with contraception based on intervention function

MoA (COM-B)	Education	Training	Persuasion	Modelling	Enablement	Incentivisation	Coercion	Environmental Restructuring	Restriction
Environmental Context and Resources (Physical opportunity)		x			✓			✓	✓
Social Influences (Social opportunity)				x	✓			✓	✓
Beliefs about Consequences (Reflective motivation)	✓		✓	x					
Knowledge (Psychological capability)	✓								
Attitude towards the behaviour (Automatic motivation and reflective motivation)	✓	x	✓	x	✓	x	x	✓	
General Attitudes / Beliefs (Themes identified in this review were linked to reflective and automatic motivation)	✓	x	✓	x	✓	x	x	✓	
Perceived susceptibility / vulnerability (Automatic motivation and reflective motivation)	✓	x	✓	x	✓	x	x	✓	
Values (Automatic motivation, reflective motivation and social opportunity)	✓	x	✓	x	✓	x	x	✓	✓

✓ = Theoretically linked and interventions identified

x = Theoretically linked but no intervention identified (missed opportunities)

The interventions were coded into five of the seven policy categories (Additional File 7): Communication/marketing (n_number of interventions_ = 8), Service provision (*n* = 8), Guidelines (*n* = 6), Environmental/social support (*n* = 2) and Regulation (*n* = 2). None of the interventions relate to the policy category “Legislation”. Based on the theoretical links between IFs and policy categories, we identified recommended policy categories that can be used to target each IF and highlighted missed opportunities (Table 4). We also identified the 26 BCTs (out of possible 74) that are theoretically linked to the key MoAs and should be used to develop behavioural change interventions (Table 5).

**Table 4:** Missed opportunities for interventions to support women with contraception based on policy categories

Intervention Function	Policy categories						
	Communication/ marketing	Guidelines	Fiscal measures	Regulation	Legislation	Environmental/ Social planning	Service provision
**Education**	✓	✓		x	x		✓
**Persuasion**	x	x		x	x		✓
**Incentivisation**	x	x	x	x	x		x
**Coercion**	x	x	x	x	x		x
**Training**		x	x	x	x		x
**Restriction**		✓		✓	x		
**Environmental restructuring**		✓	x	x	x	✓	
**Modelling**	x						x
**Enablement**		✓	x	x	x	✓	✓

✓ = Theoretically linked and interventions identified

x = Theoretically linked but no intervention identified (missed opportunities)

**Table 5:** Behaviour Change Techniques (BCTs) with theoretical links to key Mechanism of Actions (MoAs) for women accessing contraception

BCT with theoretical links	Number of MoAs
Information about health consequences	4
Information about social and environmental consequences	3
Pros and cons	3
Credible source	2
Salience of consequences	2
Social support (practical)	2
Adding objects to the environment	1
Anticipated regret	1
Avoidance/reducing exposure to cues for the behaviour	1
Biofeedback	1
Comparative imagining of future outcomes	1
Framing/reframing	1
Incentive (outcome)	1
Information about antecedents	1
Information about emotional consequences	1
Information about others’ approval	1
Instruction on how to perform behaviour	1
Material incentive (behaviour)	1
Prompts/cues	1
Remove aversive stimulus	1
Restructuring the physical environment	1
Restructuring the social environment	1
Reward (outcome)	1
Social comparison	1
Social reward	1
Social support (unspecified)	1

## Discussion

We explored barriers to and facilitators for women choosing and accessing a preferred method of contraception and examined IFs that are theoretically linked to these barriers and facilitators to make recommendations for interventions. The most frequently reported themes were: women’s personal/other people’s experience, concerns about side effects, healthcare professionals not providing sufficient information about contraception, and concerns relating to the mechanisms of the contraceptive method or the procedure of fitting them. Guided by the TaTT and BCW we identified Enablement, Environmental Restructuring, Modelling and Education as the priority IFs as they are linked with most of the commonly reported barriers and facilitators. We identified various interventions that aimed to enable women to seek help for or choose contraception and highlighted missed opportunities which can be targeted for future interventions.

The barriers and facilitators identified in this review span several MoAs and are theoretically linked to all nine IFs. Four of these IFs were considered a priority for interventions to support women in accessing contraception. One priority IF is Enablement, which is defined as increasing the means, or reducing barriers, to increase capability or opportunity (beyond training or environmental restructuring) [12]. Women reported difficulty with the accessibility of location and timing of services, such as not having a suitable appointment, or their local primary care providers not offering contraceptive services [16–19]. Some women were embarrassed to discuss issues relating to contraception [19–21], women at our public engagement also mentioned stigma around discussing contraception. In a study among homeless women in Central London, some participants reported experiencing unwelcoming healthcare settings and this discouraged them from opening up about their challenges [19], and some asylum seekers reported not being registered with a GP [22]. Women also reported that the ease of use/availability of contraception was an important factor when choosing contraception method [23, 24]. For example, many women chose condoms over other forms of contraception because they were readily available, easy to use and no preparations were required [23]. Increasing the range of locations where women can access more methods of contraception and ensuring a range of methods are easily available from the first point of access would enable more women to choose from more reliable methods of contraception. Others have discussed the possibility of making oral contraceptives available over-the-counter to reduce barriers to access [25]. This may be particularly helpful for immigrants who are yet to be registered with a GP, as well as vulnerable and marginalised women. However, this risks women not having a comprehensive discussion and may reduce the chance of them receiving the most effective long-acting methods.

Another priority IF is Environmental Restructuring which involves changing the physical and social context in which a behaviour occurs [12]. Many women expressed a preference for a female GP to discuss contraception [21, 26, 27]. Language barriers and cultural misunderstanding were reported by female Chinese asylum seekers in one study [22], and the women who participated in our public engagement. Religious beliefs also influenced women’s contraception decisions [19]. Many studies highlighted that women with learning disabilities are being excluded from decision-making, even in cases where they are capable of contributing [26–31]. Furthermore, these women are provided with information materials that are not adapted to their learning needs [26]. Similarly, in our public engagement, women reported struggling to understand existing information materials. Adaptation of existing information materials may benefit many women. This could include, for example, working with religious groups/community leaders in order to present religiously acceptable contraception options. Also, translation of information into different languages and simple English would be beneficial for non-English speaking women and women with learning disabilities. Various measures (including trusted translators, guidelines and regulations) could be put in place to ensure that these women are involved in the decision-making process at every stage.

The third priority IF is Modelling, i.e.: providing an example for people to aspire to or imitate [12]. Women frequently reported basing their choice of contraception on their personal experiences and the experiences of other women [24, 32, 33]. They are also influenced by other people’s perceived expectations of use (or non-use) of contraception and seek advice from informal sources such as mothers, friends, and women’s magazines [22, 32, 34–36]. Some women from our public engagement also expressed the influence of their male partner’s opinions on their decision-making regarding contraception. Interventions that include testimonies from women from various sociodemographic groups highlighting their lived experiences with different forms of contraception may therefore be effective. Such testimonies should address aspects that this review highlights as being important to women when making decisions about contraception (such as concerns about the procedure of fitting them). It is important to note that of the four priority IFs we identified, Modelling was the only IF for which we were not able to link any national intervention. However, we found a local organisation who hold workshops for women of colour to discuss reproductive health and contraception which may link to Modelling (Decolonising Contraception https://www.decolonisingcontraception.com). Further research on the effectiveness of modelling interventions for contraception, especially among vulnerable groups may be useful.

Education, which focuses on increasing knowledge and understanding, is the fourth priority IF [12]. Many studies report that some women have poor information/knowledge about various contraception methods [16, 20, 22, 32, 33, 36, 37]. For example, some assumed that intrauterine devices (IUDs) are “big” and were surprised when they saw a real IUD [38]. Many women are concerned about side effects of contraception, adding extra chemicals/hormones to the body or have the perception that hormonal contraceptives are unnatural [22, 23, 33, 35, 36, 39–43]. For some immigrant women, there was a lack of understanding of the UK health service context [22]. For example, in one study a participant was dissatisfied with her implant but unaware that she could see her GP to remove it and discuss alternatives [22]. Knowledge of where to obtain contraception was also important [44]. Many of these issues were also reported by the women during our public engagement. Although various interventions exist which provide education relating to contraceptives, they do not always address all the important aspects we identified, and sometimes they are not available in formats that are accessible for some women. Many information/tools are only available online whereas some women do not have internet access or lack required IT skills as highlighted by some women at our public engagement activities. Future education interventions should consider the knowledge gaps and limitations of mode of delivery identified in this review.

This review utilised a comprehensive search of three electronic databases. We engaged with key stakeholders throughout the review. We used a systematic approach involving a combination of behavioural tools, namely the TaTT and BCW, to identify important IFs and policy categories that can be targeted for behavioural interventions. Making this review specific to the UK provided valuable national context specific evidence. However, since only studies published in English and conducted in the UK were included may limit the generalisability of the findings internationally. Being a rapid review with limited time, we were not able to complete some processes in duplicate (such as data extraction, intervention coding) which would have improved the validity and reliability of the data. However, we ensured that data were sample checked by a second reviewer. Intervention descriptions were sometimes limited, which constrained our coding process. Although we conducted a comprehensive search and contacted experts to identify available national interventions, we acknowledge that this intervention search was not exhaustive. Despite this limitation, however, we believe that our search was substantial enough to ensure that all key relevant national interventions were identified. This review focused on women, but we acknowledge that those who do not identify as women, including those who are non-binary, trans-men, or gender-fluid, may face additional barriers in accessing appropriate contraception, the impacts of which warrant further exploration.

## Conclusion

We have identified many factors that influence women’s choices and access to contraception. We have highlighted four priority IFs, Modelling, Enablement, Education and Environmental Restructuring, that can be targeted to enable women to make an informed choice and access their preferred method of contraception. The existing national interventions cover three of the four priority IFs, but not Modelling. While the available national interventions cover the majority of the priority IFs, they do not address all the issues revealed in this review. This work contributed to development of a toolkit for applying behavioural science to barriers in reproductive health (available at http://wrap.warwick.ac.uk/149804/). Our findings should be considered when developing future interventions or improving existing ones.

## Supplementary Information

**Additional file 1. **Medline Search Strategy

**Additional file 2.** PRISMA Checklist

**Additional file 3. **Characteristics of included studies

**Additional file 4. **Quality Appraisal

**Additional file 5. **Quotes and Themes

**Additional file 6. **Themes, Mechanisms of Action, Intervention Functions and Behaviour Change Techniques

**Additional file 7. **Intervention Coding

## Data Availability

All data generated or analysed during this study are included in this published article [and its supplementary information files].

## Abbreviations

BCW: Behaviour Change Wheel
BCTs: Behaviour Change Techniques
COM-B: Capability Opportunity Motivation model of behaviour
FSRH: Faculty of Sexual and Reproductive Healthcare
FWT: Foleshill Women’s Training
GP: General Practitioner
IF: Intervention Function
IT: Information Technology
IUD: Intrauterine Device
MMAT: Mixed Methods Appraisal Tool
MoAs: Mechanisms of Action
NICE: National Institute for Health and Care Excellence
PHE: Public Health England
PRISMA: Preferred Reporting Items for Systematic Reviews and Meta-Analyses
TaTT: Theory and Techniques Tool
UK: United Kingdom
