# Supplementary material for: Enabling women to access preferred methods of contraception: a rapid review and behavioural analysis
Source: BMC Public Health. 2021 Nov 27;21:2176. doi: 10.1186/s12889-021-12212-7 (PMC8627100; doi:10.1186/s12889-021-12212-7)
Supplement: Supplementary file 1 — Additional file 1. Medline Search Strategy [file 12889_2021_12212_MOESM1_ESM.docx]

Additional File 1: Medline Search Strategy

Searched Ovid MEDLINE(R) ALL 1946 to October 14, 2019 on the 15^th^ of October 2019

|  | **Searches** | **Results** |
| --- | --- | --- |
| 1 | Behaviour.mp. | 195901 |
| 2 | Behavior/ | 28980 |
| 3 | exp Reproductive Behavior/ | 8765 |
| 4 | Reproductive behavior.mp. or exp Reproductive Behavior/ | 12756 |
| 5 | Choice Behavior/ | 31392 |
| 6 | Decision Making.mp. or Decision Making/ | 197998 |
| 7 | behavio* intervention*.mp. | 9950 |
| 8 | behavio* change*.mp. | 33821 |
| 9 | (behav* adj2 chang*).mp. [mp=title, abstract, original title, name of substance word, subject heading word, floating sub-heading word, keyword heading word, organism supplementary concept word, protocol supplementary concept word, rare disease supplementary concept word, unique identifier, synonyms] | 46268 |
| 10 | influence.mp. | 982197 |
| 11 | Choice.mp. | 316156 |
| 12 | Driver.mp. | 30514 |
| 13 | Facilitat*.mp. | 500057 |
| 14 | Enabl*.mp. | 411281 |
| 15 | Barrier*.mp. | 288846 |
| 16 | Decision*.mp. | 422088 |
| 17 | Help-Seeking Behavior/ or Help seek*.mp. | 5729 |
| 18 | Help-Seek*.mp. | 5729 |
| 19 | (seek* adj2 help).mp. [mp=title, abstract, original title, name of substance word, subject heading word, floating sub-heading word, keyword heading word, organism supplementary concept word, protocol supplementary concept word, rare disease supplementary concept word, unique identifier, synonyms] | 10147 |
| 20 | (seek* adj2 treatment).mp. [mp=title, abstract, original title, name of substance word, subject heading word, floating sub-heading word, keyword heading word, organism supplementary concept word, protocol supplementary concept word, rare disease supplementary concept word, unique identifier, synonyms] | 12193 |
| 21 | (seek* adj2 behavio*).mp. [mp=title, abstract, original title, name of substance word, subject heading word, floating sub-heading word, keyword heading word, organism supplementary concept word, protocol supplementary concept word, rare disease supplementary concept word, unique identifier, synonyms] | 14488 |
| 22 | (seek* adj2 health service*).mp. [mp=title, abstract, original title, name of substance word, subject heading word, floating sub-heading word, keyword heading word, organism supplementary concept word, protocol supplementary concept word, rare disease supplementary concept word, unique identifier, synonyms] | 402 |
| 23 | health seek*.mp. | 2592 |
| 24 | (seek* adj2 knowledge).mp. [mp=title, abstract, original title, name of substance word, subject heading word, floating sub-heading word, keyword heading word, organism supplementary concept word, protocol supplementary concept word, rare disease supplementary concept word, unique identifier, synonyms] | 337 |
| 25 | (seek* adj2 information).mp. [mp=title, abstract, original title, name of substance word, subject heading word, floating sub-heading word, keyword heading word, organism supplementary concept word, protocol supplementary concept word, rare disease supplementary concept word, unique identifier, synonyms] | 6147 |
| 26 | Health service*.mp. | 423190 |
| 27 | service us*.mp. | 12371 |
| 28 | service utili*.mp. | 5816 |
| 29 | care utili*.mp. | 9761 |
| 30 | health utili*.mp. | 2499 |
| 31 | treatment utili*.mp. | 1344 |
| 32 | self help.mp. | 18445 |
| 33 | self-help.mp. | 18445 |
| 34 | self-care.mp. or Self Care/ | 42116 |
| 35 | self care.mp. | 42116 |
| 36 | Healthcare.mp. | 217649 |
| 37 | Health care.mp. | 758742 |
| 38 | Implementation.mp. | 227953 |
| 39 | 1 or 2 or 3 or 4 or 5 or 6 or 7 or 8 or 9 or 10 or 11 or 12 or 13 or 14 or 15 or 16 or 17 or 18 or 19 or 20 or 21 or 22 or 23 or 24 or 25 or 26 or 27 or 28 or 29 or 30 or 31 or 32 or 33 or 34 or 35 or 36 or 37 or 38 | 4010501 |
| 40 | exp Great Britain/ | 356952 |
| 41 | (national health service* or nhs*).ti,ab,in. | 179491 |
| 42 | (english not ((published or publication* or translat* or written or language* or speak* or literature or citation*) adj5 english)).ti,ab. | 92674 |
| 43 | (gb or "g.b." or britain* or (british* not "british columbia") or uk or "u.k." or united kingdom* or (england* not "new england") or northern ireland* or northern irish* or scotland* or scottish* or ((wales or "south wales") not "new south wales") or welsh*).ti,ab,jw,in. | 1971098 |
| 44 | (bath or "bath's" or ((birmingham not alabama*) or ("birmingham's" not alabama*) or bradford or "bradford's" or brighton or "brighton's" or bristol or "bristol's" or carlisle* or "carlisle's" or (cambridge not (massachusetts* or boston* or harvard*)) or ("cambridge's" not (massachusetts* or boston* or harvard*)) or (canterbury not zealand*) or ("canterbury's" not zealand*) or chelmsford or "chelmsford's" or chester or "chester's" or chichester or "chichester's" or coventry or "coventry's" or derby or "derby's" or (durham not (carolina* or nc)) or ("durham's" not (carolina* or nc)) or ely or "ely's" or exeter or "exeter's" or gloucester or "gloucester's" or hereford or "hereford's" or hull or "hull's" or lancaster or "lancaster's" or leeds* or leicester or "leicester's" or (lincoln not nebraska*) or ("lincoln's" not nebraska*) or (liverpool not (new south wales* or nsw)) or ("liverpool's" not (new south wales* or nsw)) or ((london not (ontario* or ont or toronto*)) or ("london's" not (ontario* or ont or toronto*)) or manchester or "manchester's" or (newcastle not (new south wales* or nsw)) or ("newcastle's" not (new south wales* or nsw)) or norwich or "norwich's" or nottingham or "nottingham's" or oxford or "oxford's" or peterborough or "peterborough's" or plymouth or "plymouth's" or portsmouth or "portsmouth's" or preston or "preston's" or ripon or "ripon's" or salford or "salford's" or salisbury or "salisbury's" or sheffield or "sheffield's" or southampton or "southampton's" or st albans or stoke or "stoke's" or sunderland or "sunderland's" or truro or "truro's" or wakefield or "wakefield's" or wells or westminster or "westminster's" or winchester or "winchester's" or wolverhampton or "wolverhampton's" or (worcester not (massachusetts* or boston* or harvard*)) or ("worcester's" not (massachusetts* or boston* or harvard*)) or (york not ("new york*" or ny or ontario* or ont or toronto*)) or ("york's" not ("new york*" or ny or ontario* or ont or toronto*))))).ti,ab,in. | 1324916 |
| 45 | (bangor or "bangor's" or cardiff or "cardiff's" or newport or "newport's" or st asaph or "st asaph's" or st davids or swansea or "swansea's").ti,ab,in. | 51732 |
| 46 | (aberdeen or "aberdeen's" or dundee or "dundee's" or edinburgh or "edinburgh's" or glasgow or "glasgow's" or inverness or (perth not australia*) or ("perth's" not australia*) or stirling or "stirling's").ti,ab,in. | 197477 |
| 47 | (armagh or "armagh's" or belfast or "belfast's" or lisburn or "lisburn's" or londonderry or "londonderry's" or derry or "derry's" or newry or "newry's").ti,ab,in. | 24348 |
| 48 | 40 or 41 or 42 or 43 or 44 or 45 or 46 or 47 | 2539367 |
| 49 | (exp africa/ or exp americas/ or exp antarctic regions/ or exp arctic regions/ or exp asia/ or exp oceania/) not (exp great britain/ or europe/) | 2759418 |
| 50 | 48 not 49 | 2400304 |
| 51 | Contraception.mp. or exp Contraception/ | 53160 |
| 52 | Contraceptive*.mp. or Contraceptive Agents, Female/ or Contraceptive Devices, Female/ | 74417 |
| 53 | Birth control.mp. | 5160 |
| 54 | Pregnancy prevention.mp. | 1307 |
| 55 | Family planning.mp. or Family Planning Services/ | 48147 |
| 56 | Family Planning Policy.mp. or Family Planning Policy/ | 1872 |
| 57 | Unplanned pregnancy.mp. or Pregnancy, Unplanned/ | 2757 |
| 58 | Unwanted pregnancy.mp. or Pregnancy, Unwanted/ | 3830 |
| 59 | Unintended pregnancy.mp. | 2607 |
| 60 | (pregnancy adj1 prevent*).mp. [mp=title, abstract, original title, name of substance word, subject heading word, floating sub-heading word, keyword heading word, organism supplementary concept word, protocol supplementary concept word, rare disease supplementary concept word, unique identifier, synonyms] | 2822 |
| 61 | Abortion.mp. or Abortion, Induced/ | 85685 |
| 62 | (pregnancy adj2 terminat*).mp. [mp=title, abstract, original title, name of substance word, subject heading word, floating sub-heading word, keyword heading word, organism supplementary concept word, protocol supplementary concept word, rare disease supplementary concept word, unique identifier, synonyms] | 10586 |
| 63 | 51 or 52 or 53 or 54 or 55 or 56 or 57 or 58 or 59 or 60 or 61 or 62 | 192486 |
| 64 | 39 and 63 | 52526 |
| 65 | 50 and 64 | 9972 |
| 66 | limit 65 to yr="2009 -Current" | 1596 |
| 67 | limit 66 to english language | 1574 |
