# Supplementary material for: Enabling women to access preferred methods of contraception: a rapid review and behavioural analysis
Source: BMC Public Health. 2021 Nov 27;21:2176. doi: 10.1186/s12889-021-12212-7 (PMC8627100; doi:10.1186/s12889-021-12212-7)
Supplement: Supplementary file 3 — Additional file 3. Characteristics of included studies [file 12889_2021_12212_MOESM3_ESM.docx]

| Additional File 3: Characteristics of included studies reporting barriers and facilitators to women’s choice and use of appropriate methods of contraception or barriers to and facilitators for HCPs supporting women with these choices | | | | | |
| --- | --- | --- | --- | --- | --- |
| **Study ID (Author, Year)** | **Study design** | **Aims** | **Dates of data collection** | **Setting (including location)** | **Characteristics of study population** |
| Bexhell 2016 | Cross-sectional survey; Questionnaire; Descriptive | To assess the proportion of unplanned pregnancies among women attending antenatal clinics and those undergoing induced abortion (IA).  To assess both their previous contraceptive use and contraceptive intention, with particular focus on the use or consideration of any long acting reversible contraceptives in order to inform service redesign. | June 2013 and November 2014 | An antenatal clinic (ANC) and a gynaecology clinic in Hull and East Riding | 1649 (648 women undergoing induced abortion and 1001 women booking at antenatal clinics).  Women age 16 and over. |
| Bharadwaj 2011 | Qualitative; Semi-structured interviews; Grounded theory thematic analysis | To explore key factors inﬂuencing young women when choosing between emergency contraceptive pill and an intrauterine device. | Not reported | Community sexual and reproductive health service in north London | 26 women under age 27 seeking emergency contraception |
| Bracken 2014 | Cross-sectional survey; Online questionnaire; Statistical analysis | To identify factors involved in women’s decisions to choose particular contraceptive methods and more speciﬁcally, incentives and disincentives to use three long acting reversible contraceptive (LARC) methods: injectables, implants, and intrauterine devices/systems (IUDs/IUSs). | December 2012-March 2013 | General population (people living in the UK) | 502 women aged 18-30. Mean age of respondents 20.6 years (SD 2.1) |
| Brown 2010 | Qualitative; In-depth interview; Analysed in accordance with principles of grounded theory | To investigate the causes of unintended pregnancies and, in particular, reasons for non-use of contraception | February and July 2007 | Gynaecology Day Unit in the North of England | 24 women (aged 16 - 20) attending a Gynaecology Day Unit (GDU) for surgical abortion. |
| Earle 2012 | Cross-sectional survey; online questionnaire; Descriptive and thematic analysis | To find out who makes decisions about contraceptive use and how they are made | Not reported | Recruitment via online mailing lists (UK wide) that concern issues of disability and/or reproductive health. | 90 carers of women with learning disabilities |
| Edelman 2013 | Qualitative; Unstructured interview; Thematic analysis | To identify and understand barriers to sexual health service access. | Not reported | Hastings and Rother area on England’s South Coast | 20 women with drug problems aged over 18 years |
| Gallagher 2019 | Mixed methods; Self-administered questionnaires, interviews and focus group discussion; Descriptive for quantitative and thematic analysis for qualitative analysis | To evaluate antenatal contraceptive counselling and provision of postpartum contraception on contraceptive choices of first-time teenage mothers enrolled with a Family Nurse Partnership (FNP) programme | Not reported | Cohort of Family Nurse Partnership (FNP) clients (n=195, not all responded), Scotland, UK | 195 first time teenage mothers who are family nurse partnership clients.  85% of sample British, 4% Eastern/ Central European, 1% African, 1% Caribbean, 9% other. The majority of the sample (56%) aged 19-20. |
| Gatiss 2009 | Cross-sectional survey; Questionnaire; Descriptive. | To inform the provision of a contraception and sexual health service for women with cystic fibrosis (CF).  To study the sexual and reproductive history, current usage of contraception, the sources and quality of advice received, and particular needs of women over 16 years of age attending a regional CF centre. | Not reported | A regional CF centre | 42 women with CF. Mean age 29.7 years (range 16-51), 69% were nulliparous. |
| Gray, 2009 | Postal survey; Comparative case study;  Statistical analysis | To compare patterns of population sexual health service use and preference in areas with and without one-stop shop services (Integrated approach with services for contraception and sexually transmitted infections), as well as the effect of one-stop shops on these service choices | January to August 2005 | Young person's dedicated service in Seaside town; mainstream sexual health service in Greater London and General practice in Inner city | 3101 General population  Age 16 – 44 years |
| Hoggart 2013 | Qualitative; In-depth interviews; Thematic analysis | To understand why young women under 24 request early removal of subdermal contraceptive implant and to understand what might help them stick with it. | Not reported. | Four contraceptive and sexual health clinics in London | 20 women who have had their implant removed within a year of insertion aged 16-23 (mean 18) |
| Johnson, 2013 | Cross-sectional; Online survey (Closed and open ended questions ); Statistical | To examine the use and awareness of contraceptive methods in USA, UK, Germany, Italy and Spain | 2010 | Multinational study (UK, Germany, Spain, Italy, USA) | UK (n=510) Women aged 25-44 years who were not known to suffer from infertility. Average age 35.0 (5.6). |
| Ledger, 2016 | Mixed methods; Online survey (combination of multiple choice and open-ended questions); Descriptive and thematic analysis | To explore contraceptive decision-making for women with learning disabilities. | April - June 2012 | Recruitment via online mailing lists and personal contacts | 90 women with learning disabilities (surveys undertaken by third parties including family members, advocates and health and social care practitioners) |
| Lopez_del Burgoet al, 2013 | Cross-sectional study; Questionaires; Statistical | To assess women's attitudes towards the mechanism of action of birth control methods | October 2008-December 2008 | Multinational study- Germany, France, UK, Sweden, and Romania | 1137 (UK 203) women who were not pregnant or trying to get pregnant, or sterile. Women aged 18-29 years (28.1%), 30-39 years (40.4%), 40-49 years (31.5%)  Parity 0 (29.6%), 1+ (70.4%) |
| McCarthy, 2009 | Qualitative study; semi-structured in-depth interview; Multi-staged narrative analysis | To investigate what women with intellectual disabilities who were using contraception understood about what had been prescribed to them, and how they felt they had been treated when they sought medical help | 2004-2006 | Two counties in South East England | 23 Women with intellectual disability who (i) had current or recent experience of using contraception, (ii) were living in community-based settings and (iii) were willing and able to discuss their experiences. Age range 20-51 years. 19 were White British, 2 Black British, 1 Mauritian and 1 Southern European. |
| McDaid, 2010 | Secondary analysis of data from a cluster randomised trial; Questionnaires; Statistical analysis. | To examine contraceptive use among a particularly vulnerable subgroup: girls who reported having had sex with more than one partner by age 16 years | 1996-2000 | 25 secondary schools, Scotland | 435 sexually active girls aged 15/16 who have had more than one partner |
| Okpo, 2014 | Qualitative; Individual and small group interviews; Thematic analysis | To explore perceptions of LARC amongst young women in Grampian (Scotland) where termination rates are higher than average in order to develop a strategy to increase LARC usage. | Not reported | Public spaces (High streets and community shopping areas), Grampian, Scotland | 65 women aged 16-24. 30 (46%) were aged 20-24 years, 19 (29%) were aged 16-17 years and 16 (25%) were aged 18-19 years |
| Pownall, 2012 | Mixed methods; Cross sectional questionnaire and structured Interviews; Statistical | To compare views, attitudes and behaviours about adolescent's sexual development between mothers of young people with intellectual disability and mothers of young people without intellectual disability. | N/A | Glasgow | 60 mothers of young people (16-24 years) with mild intellectual disability (ID) and without intellectual disability. ID GROUP Mothers age 48.8 (6.5) Children's age 18.8(1.9). No ID GROUP Mothers age 48.3 (4.9) Children's age 18.0 (1.7) |
| Ramachandran, 2011 | Audit; Case notes; Descriptive statistics. | Audit of the contraception service offered to teenagers attending Scottish genitourinary medicine clinics in 2007 against various standards. The main outcomes audited for were the proportion of women who had an assessment of their need for contraception and the proportion of women in need of contraceptive advice who received it at the attendance | January to June 2007 | Main clinics of 11 Scottish health boards (Ayrshire and Arran, Grampian, Borders, Dumfries and Galloway, Lothian, Fife, Greater Glasgow and Clyde, Highland, Lanarkshire, Forth Valley and Tayside) | 579 females aged less than 18 years. |
| Roderique-Davies; 2016 | Questionnaire; cross sectional questionnaire; Multiple regression analyses. | To explore women’s intention to use long-acting reversible contraception using two established models of health behaviour: the theory of planned behaviour and the health belief model | Not reported | Eight community contraception and sexual health clinics in South East Wales | 128 women attending community contraception and sexual health centres in South East Wales. Mean age: 26.12 years, standard deviation (SD): 7.91). Range 16-45. |
| Rosales, 2012 | Cross-sectional study; Questionnaire; Statistical. | To establish whether current contraceptive choice corresponds with the qualities identified by women as important when considering a method in a primary care setting.  To investigate whether a woman’s age and past history of abortion affects her contraceptive choice. | Not reported | 2 primary care settings in North East England. | 177 English speaking women. Mean age 28.84 years; Range 14-54 years |
| Say 2009 | Mixed methods; self-completion questionnaire; Statistical analysis and thematic analysis. | To explore young people’s attitudes towards combined vaginal ring, combined patch and single rod implant contraceptives in order to develop understanding of the choices they make about contraception. | Not reported | Three young people’s contraception and sexual health clinics in Newcastle upon Tyne | 127 Women aged 13-21 years, Mean 16.62 |
| Shah 2019 | Qualitative; Semi structured face to face interviews; Thematic analysis | To gain a clearer understanding of the attitudes of homeless women towards contraception in central London. | February - March 2016 | Two homeless shelters in Central London | 14 homeless women, aged 21- 53 years, mean age 27 |
| Shawe 2011 | Mixed methods; Questionnaires and semi-structured interviews; Thematic for qualitative, descriptive for quantitative. | To understand factors which promote or discourage use of contraception in women with diabetes mellitus (DM). To identify contraceptive knowledge, skills and attitudes of health professionals involved in diabetes care. | Not stated | Four NHS trusts in South of England | 107 completed questionnaires, 16 face to face interviews with women with diabetes, 16 interviews with HCPs who specialise in diabetes. Mean age of women completing questionnaire was 31. Women selected for interview: three aged 16 – 19; four aged 20 – 29 and five aged 30 – 39, four unknown age). |
| Skouby 2010 | Structured interview; Statistical | To investigate contraceptive use and behaviour of women in 5 European countries. | January - May 2003 | Five European countries: France, Germany, Spain, Italy and UK. General population. | 12,138 participants; 2341 (19%) from UK . Randomly selected women age 15-49. |
| Thomas 2014 | Cross-sectional; Questionnaires; Univariate and multivariate logistic regression analyses | To explore recent condom use and future use intentions in community settings amongst low-risk populations | Not reported | Primary care/family planning clinics/university campus healthcare clinics | 311 known condom users in established relationships. The majority (78%) were female, and 73% were in relationships of more than a year. The mean age was 27 (range: 16–59, SD: 8.6). |
| Verran 2015 | Qualitative; Semi structured interviews; Thematic analysis | To examine how Chinese asylum seekers experience and make decisions about family planning in the UK, and how this is shaped by their cultural background. | Not reported | Specialist baby clinic for asylum seekers | 10 asylum seeking women from mainland China. Age 26- 41 years. |
| Walker 2012 | Qualitative; semi-structured interviews conducted with questionnaire participants; Interpretive phenomenology | To explore contraceptive attitudes and experiences amongst contraceptive users in the UK | 2006 - 2008 | Recruitement:: a family planning clinic, advertisement on two university websites, and by approaches to workplaces and social groups in the are in. East of England. | 16 women (+18 men). Contraceptive users in the UK. The interviewees were aged between 18 and 56 years (mean = 29.4 years). |
| Walker 2016 | Mixed methods; interviews and questionnaire; Thematic analysis of interviews, statistical analysis of questionnaire. | To examine the barriers to uptake of intrauterine contraception (IUC) by women in a GP setting in the UK. | Feb-August 2015 (for survey, not reported for interviews) | GP practices in England | 30 women for interview; 1195 women aged 18-49 for survey (mean age 33.9 years); 79.8% self-identified as White British. |
| Walmsley 2016 | Qualitative; Interviews and Focus Group discussion; Not stated | To explore the experience of women with learning disabilities and their experience of making decisions about contraception | Not stated | Not stated | 19 women with learning difficulties. |
| Williams 2014 | Cross-sectional; Researcher administered Questionnaire; Descriptive statistics | To explore the views of Sexual health services and support of younger adults with intellectual disability (ID) | Not reported | Lothian, Scotland. ID service setting. | 34 males and females. 16-35 year olds, |
| Williamson 2009 | Qualitative; in-depth interviews; Not reported but appears to be thematic analysis. | To explore the extent to which condom use has become normalised among young women. | Not reported | Recruited from a randomised trial of a school-based sex education intervention (SHARE) in Glasgow. | 20 sexually active women aged 20. |
| Williamson 2009 (2) | Qualitative; in-depth interviews; Framework analysis. | To describe young women’s accounts of hormonal contraceptive use to explore why so many rely on the contraceptive pill. | January–June 2003 and January–May 2004 | Recruited from a randomised trial of a school-based sex education intervention (SHARE) in Glasgow. | 20 women aged 16-24. |
